# The Provision of Matrix Support for the Care of Individuals With Mental Health Needs Resulting From the Use of Alcohol and Other Drugs

**DOI:** 10.1111/inm.70052

**Published:** 2025-04-15

**Authors:** Guilherme Correa Barbosa, Ricardo da Silva de Jesus, Silvia Cristina Mangini Bocchi, Thiago da Silva Domingos, Tatiane Carolina Martins Machado Rodrigues, Jaciane Araújo Cavalcante

**Affiliations:** ^1^ Nursing Department, Botucatu, Medical School São Paulo State University (UNESP) São Paulo Brazil; ^2^ Federal University of São Paulo Paulista School of Nursing São Paulo Brazil

**Keywords:** health human resource training, mental health assistance, mental health services, primary health care, substance‐related disorders

## Abstract

This study presents the findings of a workshop designed to train health professionals from the Psychosocial Care Network in matrix support for alcohol and other drug care. This experience report is based on action research and involved 45 health professionals from a municipality in northern Brazil. The workshop consisted of three interdependent meetings, with activities structured around the Constructivist Spiral framework. The meetings explored participants' perceptions of individuals who use substances and examined the technical and relational skills necessary for care delivery, teamwork and the coordination of intra‐ and inter‐sectoral strategies. The evaluations indicated that the activities were relevant and applicable to clinical practice, addressing gaps identified by participants in their healthcare settings. The workshop was effective in raising awareness and training healthcare professionals in the care of people with substance use disorders. Implementing matrix support strategies in primary care strengthens mental health services, aligning professional training with a networked approach to care.

## 1 Introduction

The field of mental health has undergone significant transformations over time, driven by the Brazilian Psychiatric Reform, which seeks to replace the asylum‐based model with a community‐ and territory‐centered approach (Ministério da Saúde 2013). From a legal and political standpoint, Federal Law No. 10.216/2001, one of the key milestones of the Psychiatric Reform, guarantees the rights of individuals with mental disorders and establishes the legal foundation for deinstitutionalisation and the development of a network of substitute services, with Psychosocial Care Centers (PCCs) serving as a cornerstone of this structure (Brasil 2001; Ministério da Saúde 2013).

Within this framework, the Psychosocial Care Network (PCN) was established to organise mental health services in an integrated and coordinated manner within the Unified Health System (UHS), using the territory and community as fundamental guidelines for care provision (Brasil 2011). Primary health care (PHC) plays a central role within PCN, characterised by its attributes of first contact, continuity, comprehensiveness and care coordination. However, challenges persist at the interface between these services, often hindering access to and continuity of mental health care.

One strategy to address these challenges is matrix support, a shared‐care management model that originated in the public health system of Campinas, São Paulo, in the late 1980s. Matrix support fosters co‐responsibility and interdisciplinary dialogue, counteracting the traditional practice of merely transferring cases to specialised services—a recurring issue in the hierarchical structure of health systems (Gonzaga and Ferreira 2017; Brasil 2011; Vilaça Mendes 2010). The fragmentation of care, combined with inadequate communication between different teams and services, results in an excessive number of referrals to specialised care, often without a thorough assessment of users' actual needs (Chiaverini 2022).

These challenges become even more pronounced in the care of individuals with needs related to alcohol and other substance use. PHC professionals frequently report difficulties in providing adequate care for this population, which compromises essential actions such as reception, screening, initial management and ongoing follow‐up. This lack of technical preparedness leads to a high number of referrals to Psychosocial Care Centers for Alcohol and Drugs (PCCAD), often without effective coordination between services (Caixeta et al. 2016; de Paula et al. 2015).

Given this scenario, action research emerges as a particularly suitable methodological approach, as it enables health professionals to actively participate in the training process and refine their practices through reflection and experiential learning. Unlike traditional research methods, action research allows for direct interventions in the studied context, fostering tangible improvements in professional training and in the daily work routines of mental health teams (Thiollent 2018). Consequently, matrix support is not only examined but also reinforced throughout the research process, equipping professionals with greater autonomy and competency in mental health care.

In this regard, in‐service training, particularly through workshops, plays a strategic role in implementing matrix support by facilitating the collective construction of knowledge and the expansion of professional practices. This perspective aligns with the concept of Permanent Health Education, which emphasises workplace‐based learning and the transformation of healthcare practices within the UHS (Iglesias and Avellar 2019; Vilaça Mendes 2010). By fostering spaces for dialogue and experimentation, workshops enable professionals to understand and adopt new strategies for patient management, encouraging an interdisciplinary approach and strengthening the care network.

Based on this framework, this study aims to present the findings of a workshop conducted with professionals from the PCN, designed to provide training in matrix support for alcohol‐ and drug‐related care. Through action research, this study not only examines the impact of this training but also explores how this methodological approach can contribute to transforming care practices within the mental health network.

## 2 Method

### 2.1 Study Design

This study employs an action research approach to explore the implementation of matrix support for alcohol and other drug care among health professionals within the PCN. Action research (Thiollent 2018) was chosen as it enables active participation, reflection and iterative adjustments in professional training, aligning with the Constructivist Spiral (CS) model (Lima 2016). This methodology fosters the collaborative construction of knowledge, integrating both theoretical and practical learning. The study intervention consisted of a workshop designed to train healthcare professionals in matrix support.

The research team consisted of two researchers with expertise in mental health nursing and qualitative and participatory methodology, a master's student in nursing with extensive experience in mental health care for people with substance use disorders and two undergraduate nursing students who were properly trained to participate in the workshops.

### 2.2 Study Setting

The research was conducted in Gurupi, a city in Tocantins, northern Brazil, with an estimated population of 88 428. The local PCN consists of 50 PHC Units, of which 16 operate under the traditional model, while 34 follow the Family Health Strategy model. Additionally, the network includes two PCC: one Type I and one Type III for alcohol and drug use disorders.

The difference between PCC Type I and PCC Type III lies in their target population and scope of care. PCC Type I provides treatment for individuals of all ages with psychiatric and substance use disorders. In contrast, PCC Type III specialises in substance use disorders and offers 24‐h inpatient care, 7 days a week.

The study targeted health professionals from three strategically selected Basic Health Units located in the north, south and central regions of the municipality. These units were chosen based on their weaker integration with PCCAD‐Type III, with the objective of fostering future matrix support, strengthening interprofessional collaboration and facilitating referrals and periodic meetings.

### 2.3 Participants

The study population included health professionals from the selected Basic Health Units and PCCAD‐Type III. Participants were recruited in person at their workplaces by the first author, ensuring direct engagement with potential participants.

Participants were eligible if they were permanent or contracted municipal employees, had worked for at least 6 months and expressed interest in and availability to participate. Professionals who missed two out of three workshop meetings, were on leave during data collection, or failed to complete workshop activities were excluded.

A total of 57 professionals were invited, but 12 were excluded, as 8 withdrew and 4 were on sick leave. The final sample consisted of 45 participants, including 23 community health workers, 9 nurses, 6 nursing technicians, 3 receptionists, 2 psychologists, 1 physician and 1 physiotherapist.

To ensure a collaborative learning environment, participants were consulted about the researcher assuming the role of workshop facilitator, given his professional experience at PCCAD‐Type III. All participants consented to this arrangement. There were no non‐participant observers in the study.

### 2.4 Workshop Introduction

The workshop served as the core intervention of the action research, structured according to the CS model. Conducted in February 2022, it comprised three weekly sessions, each lasting approximately 3 h. Participants were assigned to morning or afternoon groups to minimise disruptions to healthcare services.

Each session followed a structured format: a welcoming and warm‐up activity, a review of the previous session, two or three exercises aligned with the session objectives, a reflection and feedback activity, and a closing discussion. Workshop activities were designed to enhance participation and engagement, reinforcing the reflection‐action‐reflection cycle as a pedagogical strategy (Thiollent 2018; Lima 2016). This aligns with the Permanent Health Education framework, which promotes workplace‐based learning and professional transformation (Iglesias and Avellar 2019).

### 2.5 Data Collection

Data were collected using multiple qualitative methods to ensure triangulation. A sociodemographic questionnaire was applied to gather information about participants' age, sex, ethnicity, marital status, income, education level, workload, employment duration in primary healthcare and contract type. Document analysis included written materials produced by participants, such as notebooks, worksheets and conceptual maps, to assess perceptions, experiences and learning outcomes. Participant observation was conducted by two researchers who documented verbal and non‐verbal interactions, focusing on engagement and group dynamics.

Each session was audio‐ and video‐recorded, supplemented by photographs and researcher field notes. The research team consisted of five members with distinct roles: one researcher facilitated activities, two observed participant interactions and two documented discussions through notes and iconographic materials.

### 2.6 Data Analysis

Data was analysed using thematic analysis, guided by the problematization framework based on the CS model (Lima 2016). The analysis followed a structured process, including identifying problems, formulating explanations, elaborating questions, constructing new meanings, and evaluating processes and outcomes. The themes were directly aligned with the objectives of the activities proposed throughout the three workshops.

Unlike traditional saturation‐based approaches, this study employed purposeful sampling, selecting participants based on their rich experience with the problem in their daily professional practice. Given the complexity of the research topic, theoretical saturation was not applicable. Instead, the CS framework was used to ensure meaningful engagement with the data.

The research team actively participated in the analysis process, engaging in discussions to interpret the emerging themes and ensure that findings were consistent with the workshop objectives. Findings were validated through a synthesis of the workshops presented to participants in subsequent sessions. This process allowed for participant verification of the interpretations and conclusions drawn from the discussions. No software was used for data analysis; coding and interpretation were conducted manually.

#### 2.6.1 Ethical Aspects

The study was approved by the Research Ethics Committee of [Institution Name] (Protocol No. 5.082.013), following Brazilian National Health Council Resolution No. 466/2012. All participants provided written informed consent and were informed of their right to withdraw at any time. To ensure confidentiality, participant names were anonymised and personal data were stored in a password‐protected database.

In this article, Artificial Intelligence was used to assist in translation. The original text was translated from Portuguese into English using DeepL Translate, with additional language verification conducted through Open Writefull to ensure accuracy.

## 3 Results

The results are presented in a way that allows for the presentation of the experiences and expectations of health professionals from the PCN services in their work context. These are presented below.

Study participants were predominantly women (82.2%, *n* = 37), married (55.8%, *n* = 24), Catholic (54.8%, *n* = 23), aged between 23 and 59 years (mean age 40 years). In terms of educational achievement, 42.2% (*n* = 19) of the participants had obtained a higher education qualification and/or a post‐graduate degree, while 57.8% (*n* = 26) had completed secondary education. The participation of community health agents was predominant among the professional categories, representing 51.1% (*n* = 23) of the total number of participants. This was followed by nurses, who made up 20% (*n* = 9) of the sample, and nursing technicians, who made up 13.3% (*n* = 6) of the sample. The duration of employment in primary health care ranged from two to 26 years, with an average of 9.24 years. In terms of the type of employment relationship, most of the participants (64.4%, *n* = 29) were employed permanently. The duration of operation within the coverage area ranged from two to 26 years, with an average of 7.56 years.

In this study, data were collected through transcription of verbal contributions made by one of the researchers during each meeting. Following the consolidation of the data from each meeting, the context of each meeting was analysed. Subsequently, a synthesis was made based on the discussions held and the issues raised, with the objective of organising the thematic axes.

### 3.1 First Meeting: ‘Who is Pissica?’

The first meeting began with a welcoming activity, followed by the introduction of the researchers and participants, the presentation of the research project and the signing of the Informed Consent Form. The group collectively established a co‐existence contract, which included agreements and commitments to guide interactions throughout the three workshops. The objectives of this first meeting were: (1) to identify the perceptions of healthcare professionals regarding people with substance use disorders and (2) to recognise the available and absent resources in the healthcare network to care for this population.

The central activity, titled ‘Who is Pissica?’, was inspired by a local slang expression used in the region to refer to individuals facing social vulnerability and suffering due to alcohol and other drug use. By adopting this culturally rooted term, the activity sought to bring participants' experiences and social conceptions to the surface, aligning with the processes of identifying problems, formulating explanations and developing questions from the CS (Lima 2016).

At the first step of this activity, participants were invited to create a visual representation of Pissica, using magazines, brushes, glue and scissors. Divided into two small groups, professionals were asked to construct an image of a person experiencing problems with alcohol and other drugs, based on their own perceptions and experiences in the healthcare setting. The researcher‐facilitator observed participants' engagement and provided support for those who showed resistance or discomfort during the activity.

After completing their visual representations, participants engaged in a discussion to present their work, explaining the elements they incorporated and the reasoning behind their choices. This step facilitated a collective analysis of the stereotypes and social stigmas attributed to people who use alcohol and other drugs. Researcher‐observers synthesised key themes from the discussion, enabling participants to critically reflect on how these perceptions influence their professional practices and interactions with this population. Similarly, this activity enables the identification of issues, the formulation of explanations and the creation of CS questions (Lima 2016).

**FIGURE 1:**
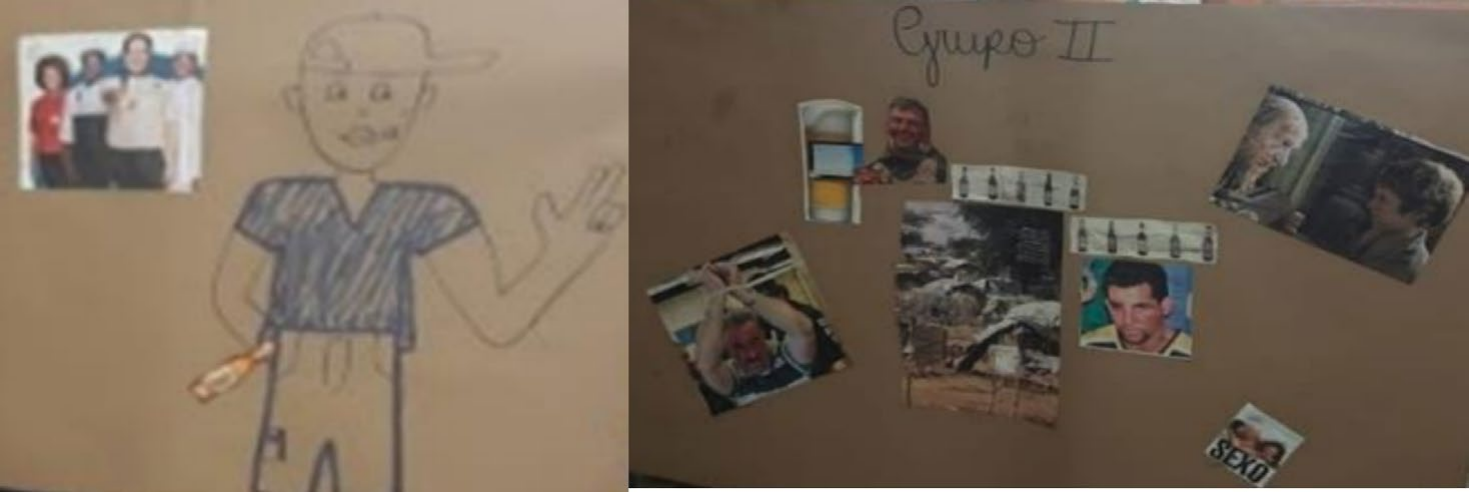
Production of the activities ‘Who is Pissica?’.

In the first group, participants constructed the image of a black man, with poor hygiene, yellow teeth, wearing jean shorts, flip‐flops (Havaianas) and a backward cap. According to the group, this individual began drinking alcohol to escape family conflicts and, 6 months ago, started using crack cocaine. The second group represented a 34‐year‐old man, dressed in dirty and torn clothes, with a foul odour and fractured teeth. The image also portrayed a moment of aggression, with the individual being physically restrained, symbolising a person in a state of imprisonment and social exclusion (Figure 1).

The representations produced by participants revealed deeply rooted stigmas and stereotypes, reinforcing the perception of people with substance use disorders as dangerous, marginalised and socially excluded individuals. These representations reflect structural prejudice and the criminalisation of substance use, reinforcing exclusionary practices in healthcare settings. This perspective fails to acknowledge that not all individuals who use alcohol or other drugs experience social or functional impairments. By equating substance use with inevitably harmful consequences, these stigmatised perceptions create barriers to care and contribute to the exclusion of individuals who could benefit from harm reduction strategies and non‐punitive healthcare approaches.

The second activity, titled ‘Hold that B.O.’, refers to an expression commonly used by health professionals in the local context, meaning ‘taking responsibility for solving a complex situation’. Furthermore, B.O. is an abbreviation for ‘Boletim de Ocorrência’, an official document used by Brazilian police departments to formally register a legally relevant complaint or incident.

In this activity, each group exchanged their visual representation of Pissica and was asked to identify the professional tools and strategies available (present) and those lacking (absent) to provide care for the person constructed by the other group. This activity once again establishes a connection between problem identification processes, explanation formulation and the creation of CS questions (Lima 2016).

Participants identified tools already present in the healthcare network, such as reception and screening, family involvement, group activities and referrals to PCCAD. However, they also pointed out significant gaps, including the absence of communication mechanisms in the health services, lack of professional training, limited management support and inadequate physical infrastructure and security. A critical issue identified was that primary care professionals did not recognise themselves as capable of managing care for people with substance use disorders, delegating this responsibility to PCCAD. This highlights the fragmentation of care within the PCN and the lack of shared responsibility among health professionals.

The evaluation was conducted through the reflection triggers: ‘How nice that…’, ‘What a shame that…’, ‘How about if…’. Participants highlighted the interaction among professionals, the exchange of knowledge and the recognition of the care network as positive aspects. However, they pointed out the limited time for discussion as a weakness. As a suggestion, they proposed extending the workshops and including additional themes related to mental health and substance use care.

### 3.2 Second Meeting: ‘Communicating with Pissica’

The second meeting began with a review of the previous workshop's productions, the presentation of the participants' evaluation and an overview of adjustments made to address the limitations identified in the first meeting. The objectives of this meeting were: (1) to reflect on communication and interaction strategies between healthcare professionals and individuals with substance use disorders; and (2) to identify effective approaches to provide comprehensive care.

The first activity, titled ‘You Don't Drink, Do You?’, aimed to encourage participants to reflect on their typical responses when interacting with people experiencing problems related to alcohol and other drug use. Divided into two smaller groups, one group was responsible for developing the character of the health professional, while the other created the character of the person with a substance use disorder. Participants were then tasked with writing a care script, depicting an interaction between these two characters and acting out the scene. This activity used role‐play technique, aligned with the CS (Lima 2016), allowing participants to identify problems, formulate hypotheses and generate new meanings through experience.

The dramatisation, titled ‘Alcoholic—Pissica: The Terror of the Basic Health Unit’, recreated a real‐life situation in a Basic Health Unit. In this case, a man under the influence of alcohol arrived at the unit with a fractured arm and abrasions. The user displayed aggressive behaviour, which led the team to call the police for support. However, one professional adopted a calm and empathetic approach, which de‐escalated the situation and established a bond between the professional and the user. The group expressed feelings of fear, insecurity and lack of preparation, stating: ‘We don't know what to do’. This reaction reflects the fragility of primary care teams when dealing with people in situations of social vulnerability and mental distress.

The second part of activity was conducted using the Debriefing technique (de Góes and Jackman 2020). Through dramatised role‐play and guided dialogue, participants reflected on their communication strategies, emotional reactions and the ethical challenges involved in providing care for people with substance use disorders. Following this, screening tools for alcohol and other drugs were presented and their applicability to PHC was discussed. This activity corresponds to the moment of constructing new meanings in the CS (Lima 2016).

The third activity, titled ‘Get on with it’, aimed to systematise the stages of initial care for people with alcohol and drug‐related problems, applicable to all professional categories. Participants worked in small groups to analyse a problem situation and complete a matrix for recording care actions (Table 1). Afterward, each group shared their production, and the researcher‐observers synthesised the results, facilitating critical reflection on practice and facilitating the construction of new meanings of CS (Lima 2016).

**TABLE 1:** Matrix for systematising the resolution of the problem situation—*‘Get on with it’ activity*—Groups 1 and 2.

Matrix questions	Group 1	Group 2
What is the step?	Multiprofessional consultation between Basic health unit and alcohol and other drugs psychosocial care centre III	Patient involvement
What is the purpose of the step?	Create a patient‐professional bond and get to know the patient's experience	The patient realises that they need help. Recognise that you are sick.
Who is responsible for the step?	Basic Health Unit and Alcohol and Other Drugs Psychosocial Care Centre III	The patient himself
Does this step have connections?	Yes	Family/Friends/Teams
Who can help with this step?	Psychologist/Psychiatrist/Nurse/Nursing Technician Community health worker/Patient's mother	Family Friends Teams
Who can get in the way?	Patient	Work environment/friends Patient/Family Conflicts
How can the step be considered complete?	When the patient accepts the treatment When there is a patient‐professional bond	When he is free of his addiction

As part of the evaluation of the processes and outcomes of the CS, participants engaged in a reflective activity using three guiding prompts: ‘In today's meeting, I learned that…’, ‘I built that…’ and ‘I felt that…’.

From these reflections, it became evident that providing effective care for people with substance use disorders cannot be done in isolation. Instead, participants recognised that collaboration and a well‐structured network are essential. The workshops illustrated that challenges in caring for this population are not exclusive to a single level of care but are present across both primary and specialised services, emphasising the need for better coordination within the healthcare system.

The term ‘link’ emerged as a recurring theme, highlighting participants' realisation that building comprehensive care requires shared responsibility and collective action. This was particularly evident in discussions about the connection between primary care teams and specialised services, such as PCCAD. Participants acknowledged that their current approach often lacked integration, reinforcing the need to strengthen networked care and interdisciplinary collaboration.

Finally, participants expressed feelings of difficulty and frustration, recognising the complexities and structural barriers that healthcare workers face at all levels. These challenges underscored the importance of continuous professional development, inter‐sectoral partnerships and supportive management strategies to improve care for people with substance use disorders.

### 3.3 Third Meeting: ‘Networks: Ideal and Real Perspectives’

The third meeting began with a reflection on the participants' emotional experiences from the previous workshops. Next, the previous productions were reviewed, and the objectives of this meeting were presented: (1) to construct a care network for people with alcohol and drug‐related issues, based on the PCN and intersectorality; and (2) to reflect on the role of PHC in matrix support for this population.

The first activity, titled ‘When One Doesn't Want It, Two Don't Fight!’, was designed to encourage participants to reflect on teamwork, communication and the challenges of collaboration within the healthcare network. The activity aimed to highlight how requests for assistance are made and received, and how professional interactions impact the effectiveness of care delivery. Aligned with the CS framework (Lima 2016), this exercise facilitated the construction of new meanings, emphasising the relational and technical integration necessary for effective teamwork.

To carry out this activity, participants were divided into two groups: active participants and observers. Those participating in the dynamic formed a circle and were instructed to throw a ball to another participant, who then passed it to another and so on, until everyone had received and thrown the ball once. The final participant would then return the ball to the first, maintaining a fixed sequence. As the activity progressed, additional balls were gradually introduced, increasing the complexity of the interactions and requiring participants to coordinate their movements and responses. In the final stage, participants were instructed to walk around the room while maintaining the sequence of ball exchanges, further challenging their ability to collaborate, focus and adapt under pressure.

At the end of the activity, participants were asked to describe their experience using a single word. Responses included ‘difficult’, ‘tiring’, ‘attention’, ‘focus’, ‘agility’, ‘perception’, ‘direction’, ‘concentration’ and ‘identification’. This was followed by a roundtable discussion, where both active participants and observers shared their reflections.

The debriefing process revealed that the exercise symbolised the healthcare network itself, with the ball representing the need for help and the act of passing it reflecting the way responsibilities are transferred within the system. Participants recognised that, in their daily practice, they often ‘pass the problem forward’ rather than taking steps to resolve it. This tendency was particularly evident in the way professionals rely on referrals to PCCAD and other specialised services, instead of assuming their role as primary care providers within the network.

Another key theme that emerged was communication. Participants discussed how clear, unambiguous communication fosters trust and improves coordination, while miscommunication can lead to misunderstandings, decreased adherence to treatment and loss of confidence from patients and their families. The exercise reinforced the importance of effective teamwork, proactive problem‐solving and shared responsibility in improving care for people with substance use disorders.

The second activity, titled ‘Networks: the Ideal, the Real, and the Possible’, aimed to examine the structure and functioning of the PCN by guiding participants through three key steps: (1) recognising existing network resources, (2) identifying gaps in care and inter‐sectoral articulation and (3) developing a diagrammatic representation of an ideal network based on the participants' perspectives.

**FIGURE 2:**
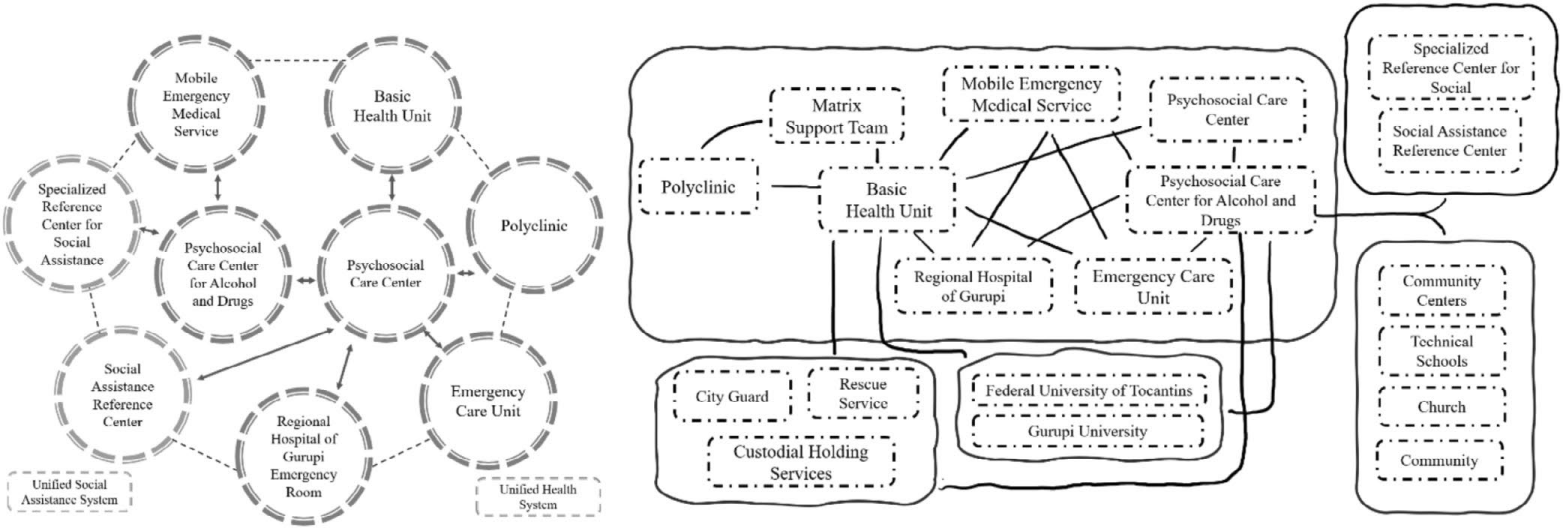
Productions of activity Networks: the ideal, the real and the possible—to the right of Team 1, to the left of Team 2.

Participants were divided into two smaller groups, each responsible for mapping the current state of the network and proposing strategies for a more effective and coordinated care system. Using visual tools, each group constructed a diagram of their proposed network, considering the integration of PHC, specialised services (such as PCCAD) and inter‐sectoral partnerships with education, public security and social assistance (Figure 2).

After presenting their diagrams, participants engaged in a debriefing session facilitated by researcher‐observers, who encouraged critical reflection on user‐centered care and the role of PHC within the PCN. Through discussion, participants acknowledged the need for stronger connections between primary care and specialised services, as well as greater coordination with other sectors to ensure comprehensive and continuous care.

A key insight that emerged was the recognition that transitioning from the ‘real’ to the ‘ideal’ network requires concrete actions within the possible reality. Participants emphasised that effective networked care depends not only on service availability but also on the proactive engagement of healthcare teams in activating resources and strengthening collaborative relationships within and beyond the health sector.

To capture participants' overall impressions of the workshop, they were invited to summarise their experience in a single word. The terms that emerged included ‘knowledge’, ‘clarification’, ‘vision’, ‘guidance’, ‘persistence’, ‘multiprofessional approach’, ‘flexibility’ and ‘courage’. These responses reflected the evolution of participants' perspectives, from an initial focus on individual cases and service limitations to a broader understanding of networked care and shared responsibility.

To conclude the workshop, a video compilation of images recorded throughout the sessions was shared with participants, providing a moment of emotional closure and reflection on the collaborative learning process.

## 4 Discussion

The objective of this study was to develop and refine a workshop methodology aimed at training professionals within the PCN in matrix support for alcohol and other drugs. The structured activities were designed to facilitate professional development, emphasising theoretical knowledge, practical application and critical reflection. The use of the CS framework, aligned with active learning strategies, proved to be a promising educational tool. This approach enabled participants to reconfigure their care practices and processes through the action‐reflection‐action cycle (de Góes and Jackman 2020; Thiollent 2018; Lima 2016).

The findings of this study are consistent with existing research on professional training in the field of mental health and substance use care. Similar studies have identified that preconceptions and stigmas among healthcare professionals act as significant barriers to effective and inclusive care (Silveira et al. 2021; Carniel et al. 2020). In this study, the critical reflection developed in the activity ‘Who is Pissica?’ demonstrated potential for transforming these perceptions, broadening healthcare professionals' understanding of individuals who use substances in various patterns—particularly those who experience severe social, occupational and financial consequences. These findings align with those of de Fo Faria et al. (2020), who reported that professionals often struggle with conceptualising the complex subject matter related to matrix support actions. Moreover, high staff turnover in mental health services has been cited as a key barrier to sustaining effective interventions, as it disrupts the continuity of care and weakens professional expertise in handling substance use disorders (de Fo Faria et al. 2020).

Expanding professionals' understanding of substance use has a direct impact on their clinical approach, influencing how they interpret the role of PHC in mental health support (Melo et al. 2018). Studies emphasise that training and raising awareness among PHC professionals is crucial to changing attitudes, thus ensuring the provision of comprehensive, person‐centred care (Silveira et al. 2021; Rossato Siqueira et al. 2019).

From a mental health nursing perspective, this study reinforces the importance of a psychosocial model of care in addressing substance use disorders. In nursing practice, the Singular Therapeutic Project plays a fundamental role, structured around the Expanded Clinic model, which emphasises individualised, multiprofessional care planning. This study supports the integration of matrix support as a tool to strengthen interprofessional collaboration, helping to overcome fragmentation in mental health services and fostering a more coordinated and person‐centred approach (da Do Nascimento Rocha and De Fátima Lucena 2018).

A systematic review of the literature highlights persistent theoretical and practical gaps in the training of mental health professionals, largely due to the historical dominance of the biomedical model in health education. This biomedical emphasis tends to reduce substance use to a purely biological issue, overlooking its social, cultural and political dimensions (Silveira et al. 2021; Carniel et al. 2020; de Fo Faria et al. 2020; Caixeta et al. 2016). Addressing these gaps requires a paradigm shift, integrating psychosocial interventions, harm reduction strategies and collaborative care models into everyday nursing practice.

The findings from this study reinforce the need for ongoing professional training, as recommended by existing research. Participants demonstrated the potential for modifying professional practices, incorporating multiprofessional consultation, structured action planning and a networking approach to improve care for individuals experiencing alcohol and other drug‐related issues (Caixeta et al. 2016; de Paula et al. 2015).

The study also identified key challenges in the establishment of interdisciplinary team care and in the integration of intra‐sectoral and inter‐sectoral networks. These findings align with the work of who argue that health work itself is a technology of care production, developed in real‐time interactions between professionals and users rather than simply following predefined protocols (de O'Cruz et al. 2020).

The workshop expanded participants' analytical capacities, leading them to recognise the need to integrate diverse services and sectors to reinforce PHC (Iglesias and Avellar 2019; Caixeta et al. 2016; de Paula et al. 2015). Moreover, the study highlights the need for technical training to equip PHC teams with the necessary skills to provide longitudinal care and health education for people with substance use disorders (Rossato Siqueira et al. 2019).

A key strength of this approach was the use of small, heterogeneous working groups, which facilitated the formation of new professional relationships, encouraged interdisciplinary dialogue and provided insight into the operations of different health services. This structure fostered a shared understanding of care gaps, leading to the co‐construction of a collective definition of the challenges faced in caring for people with alcohol and other drug‐related issues.

This collaborative problem‐solving process enabled the integration of different points within the Healthcare Network, reinforcing the importance of team‐based decision‐making and networked care (Silveira et al. 2021; Rossato Siqueira et al. 2019).

The action‐reflection‐action cycle used in this study contributed to strengthening the psychosocial model of care, challenging biomedical and moralistic perspectives on substance use. However, a key challenge remains: ensuring that the insights gained from reflective practice translate into concrete actions in daily clinical work. The CS framework provides an avenue for professionals to continuously rethink and reconstruct their practices, helping them to identify barriers and implement feasible solutions in mental health care (Rossato Siqueira et al. 2019; Thiollent 2018; Lima 2016).

Delivering care within a psychosocial and anti‐asylum framework requires dedicated time and space for professionals to process their experiences, navigate challenges and build meaningful therapeutic relationships—particularly when working with individuals in borderline situations of social vulnerability. Creating structured opportunities for discussion and critical reflection is crucial for fostering ethical, person‐centered mental health care (Carniel et al. 2020; de O'Cruz et al. 2020; Rossato Siqueira et al. 2019).

Finally, the study by Sanches and Vecchia (2020) highlights family estrangement and low family engagement in treatment as significant concerns. They emphasise that the family plays a crucial role in social protection and recovery, and that insufficient investment in psychosocial rehabilitation exacerbates marginalisation and exclusion among people who use drugs. These findings reinforce the importance of social inclusion policies and community‐based interventions in mental health care.

This study did not aim to assess knowledge acquisition or skill development among workshop participants. Instead, it sought to demonstrate the applicability of the CS as a training framework for in‐service education. The study underscores the importance of providing theoretical, practical and political support for professional training programmes focused on mental health and substance use care.

The CS has demonstrated strong potential for meaningful learning, promoting critical engagement among professionals and encouraging them to analyse their work environments through a psychosocial lens (Backes et al. 2018). Given the prevalence and impact of substance use disorders on users, health professionals and policymakers, this educational approach is both relevant and replicable across diverse healthcare settings.

## 5 Conclusion

In conclusion, the workshop successfully facilitated the training and sensitisation of healthcare professionals in the care of individuals with alcohol and other drug‐related issues. The activities encouraged a critical examination of key aspects of care, including the reception process, interpersonal relationships, teamwork, and intra‐ and inter‐sectoral collaboration. By providing structured spaces for critical reflection and professional development, the workshop strengthened participants' skills and attitudes, promoting ethical, effective and safe clinical practice within the PCN. This highlights the importance of integrating reflective training methodologies to enhance healthcare professionals' ability to respond to the complex needs of this population.

### 5.1 Relevance for Clinical Practice

This study highlights the crucial role of mental health nursing in providing comprehensive, person‐centered care for individuals with substance use disorders. The workshop methodology—grounded in action research, the CS and public health policies on alcohol and drug use care—demonstrated how active learning strategies can enhance clinical practice by fostering critical thinking, professional collaboration and ethical decision making.

For mental health nurses, these findings reinforce the importance of strengthening reception and interpersonal care in psychosocial services, ensuring that individuals with substance use disorders receive non‐judgmental, stigma‐free support. The study also emphasises the need to enhance teamwork and interdisciplinary collaboration within the PCN, promoting more effective care coordination between primary healthcare services and specialised mental health facilities.

Incorporating reflective training methodologies into mental health nursing education contributes to developing problem‐solving skills and critical thinking, enabling professionals to identify barriers in care and implement context‐sensitive, tailored interventions. Additionally, the study highlights the relevance of inter‐sectoral collaboration, recognising the impact of social determinants of health on substance use and reinforcing the role of mental health nurses in advocating for partnerships with social services, education and public security sectors.

By integrating participatory training methodologies into mental health nursing education and in‐service training, this study reinforces the need for ongoing professional development to equip nurses with the knowledge, skills and attitudes necessary to provide holistic, recovery‐oriented care to individuals experiencing alcohol and other drug‐related problems.

## Conflicts of Interest

The authors declare no conflicts of interest.

## Data Availability

The data that support the findings of this study are available from the corresponding author upon reasonable request.
